# Nonlinear association of the systemic immune-inflammatory index with mortality in diabetic patients

**DOI:** 10.3389/fendo.2024.1334811

**Published:** 2024-02-13

**Authors:** Chunli Meng, Kai Liu

**Affiliations:** Center of Infectious Diseases, West China Hospital, Sichuan University, Chengdu, China

**Keywords:** systemic immune-inflammation index, all-cause mortality, cardiovascular mortality, diabetes, neutrophil, lymphocyte

## Abstract

**Background:**

It has been demonstrated that in diabetic patients, an elevated neutrophil-lymphocyte ratio (NLR) is independently connected with higher cardiovascular and all-cause mortality. It is unclear, however, if the systemic immune-inflammatory index (SII) and the mortality rate among diabetic patients are related. Investigating the linkage between SII and diabetes patients’ risk of cardiovascular and all-cause death was the aim of the study.

**Methods:**

4972 diabetics who were chosen from six rounds of the National Health and Nutrition Examination Survey (NHANES) between 2005 and 2016 were the study’s participants. The optimal SII threshold with the highest correlation with survival outcomes was identified by applying the Maximum Selection Ranking Statistical Method (MSRSM). To assess the relationship between SII and cardiovascular and all-cause mortality in diabetics, subgroup analysis and Cox regression modeling were employed. Furthermore, smoothed curve fitting was utilized to determine the nonlinear relationship of them.

**Results:**

Over the course of a median follow-up of 69 months (interquartile range [IQR], 54-123 months), 1,172 (23.6%) of the 4,972 diabetic patients passed away. These deaths included 332 (6.7%) cardiovascular deaths and 840 (16.9%) non-cardiovascular deaths. Individuals were categorized into higher (>983.5714) and lower (≤983.5714) SII groups according to MSRSM. In multi-variable adjusted models, subjects with higher SII had a significantly increased chance of dying from cardiovascular disease (HR 2.05; 95% confidence interval (CI):1.42,2.97) and from all causes (HR 1.60; 95% CI:1.22,1.99). Kplan-Meier curves showed similar results. Subgroup studies based on age, sex, BMI, drinking, smoking, and hypertension revealed that the connection maintained intact. The previously stated variables and SII did not significantly interact (p interaction > 0.05). In diabetic patients, smooth curve fitting revealed a nonlinear correlation between SII and mortality.

**Conclusion:**

In diabetic patients, elevated SII is linked to higher cardiovascular and all-cause mortality.

## 1 Introduction

Diabetes, one of the most serious and common chronic disorders in modern times, shortens life expectancy and can have disastrous, costly, debilitating, and even deadly effects (1, 2). It has also become a major public health issue (3). In addition, diabetes can lead to microvascular complications (such as nephropathy, retinopathy, neuropathy and so on) as well as macrovascular complications (such as coronary artery disease, stroke, and peripheral vascular disease and so on), which can reduce the patient’s quality of life (4). The 10th edition of the International Diabetes Federation (IDF) Diabetes Atlas predicts that among people aged 20 to 79 years, 10.5% (or 536.6 million) would have diabetes globally by 2021. It is estimated that by 2045, that percentage will rise to 12.2% (783.2 million) instances (5). Research has demonstrated that individuals with diabetes mellitus bear a notably elevated risk of dying from cardiovascular disease(CVD) and other causes (6, 7). Thus, it’s critical to promptly identify new risk factors in order to stop, impede, or lessen the development of diabetes and the number of deaths associated with the disease.

The systemic immune-inflammation index (SII), a complete new immune-inflammation biomarker was got through platelet, neutrophil, and lymphocyte counts, was created by Hu et al. It accurately depicts both the local immune response and the body’s systemic inflammatory condition (8). One of the factors contributing to obesity and insulin resistance in obese human patients and animal models (such as db/db mice) has been suggested to be systemic inflammation (9, 10). Diabetes and low-grade systemic inflammation have been shown to be related (11). The SII was used to evaluate the prognosis of patients suffering from a variety of malignancies initially, such as non-small cell lung cancer, stomach cancer, and rectal cancer (8, 12–14). However, it has recently been discovered that SII is positively correlated with the incidence of comorbidities and sequelae, including diabetic nephropathy and depression, in individuals with diabetes and so on (15, 16). Furthermore, research has demonstrated that elevated levels of SII are highly predictive of cardiovascular disease (CVD) and all-cause mortality in the general individuals (17). The relationship between SII and the prevalence of diabetes has been clarified (18). Studies have also been conducted on the relationship between SII and mortality in diabetic patients. The study by Wei Q et al. (19) showed a U-shaped correlation between SII and all-cause mortality and a J-shaped association with CVD mortality. And the study of Liu Y et al. (20) showed a U-shaped relationship between SII and all-cause mortality in diabetic, prediabetic and non-diabetic populations, and a non-linear relationship between SII and CVD mortality in prediabetic and non-diabetic populations. However, in diabetic patients, there was a linear relationship between SII and cardiovascular mortality. The relationship between SII and mortality in diabetic patients remains unclear.

Thus, we examined the linkage between SII and the risk of cardiovascular and all-cause death in a sizable, nationally representative patient cohort with diabetes.

## 2 Materials and methods

### 2.1 Data source and study population

**Figure 1:**
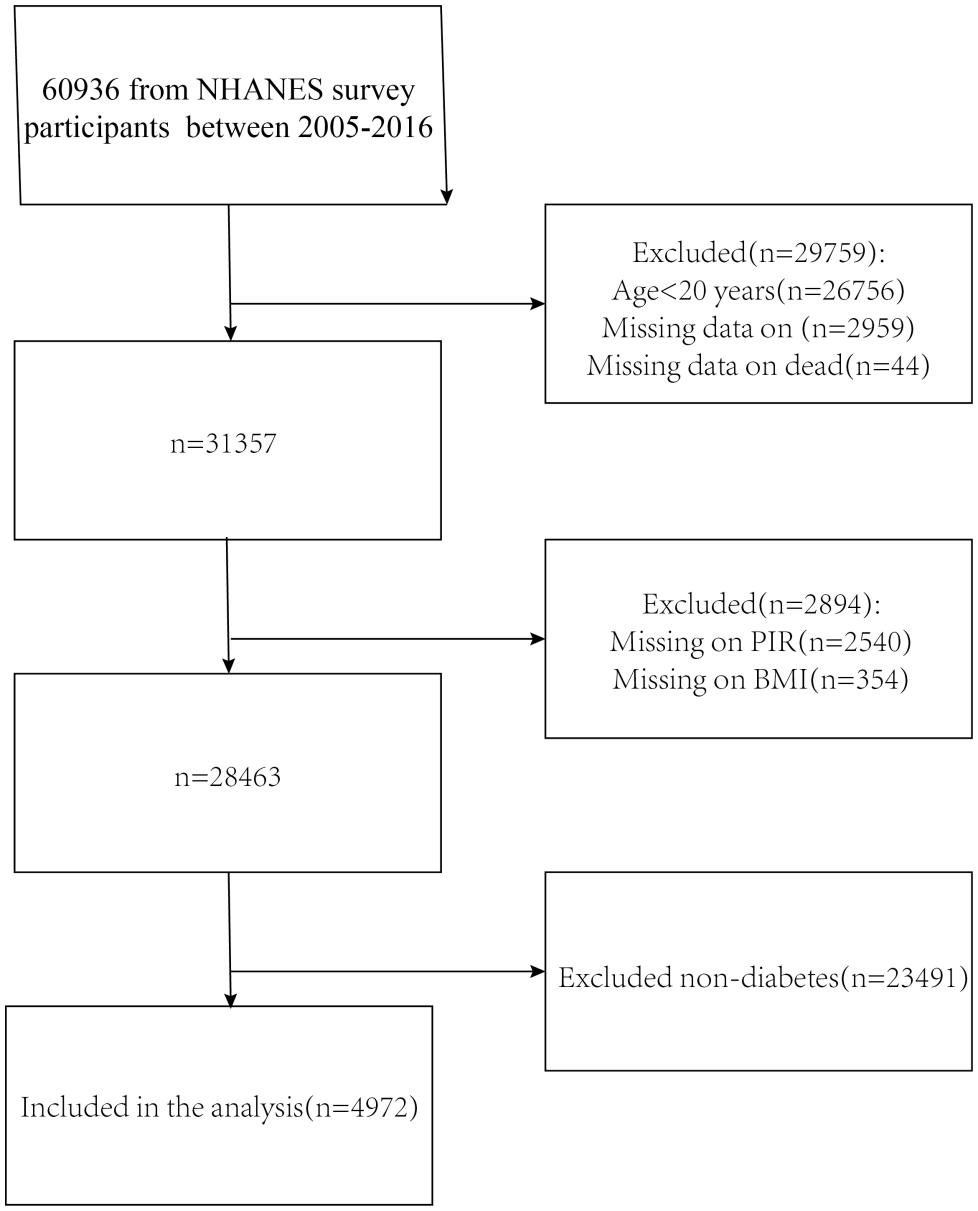
Flowchart for choosing participants. National Health and Nutrition Examination Survey, or NHANES.

The NHANES is a comprehensive research study that assesses the nutritional and physical health of adults and children in the United States. The NCHS Research Ethics Review Board gave it official approval, and each study participant completed an informed consent form. This study included data from six NHANES cycles (2005-2016) with a total of 60,936 participants. We removed respondents under the age of 20, as well as those who lacked complete survival and laboratory test information,eventually, 4,972 people were enrolled in this study (**Figure 1**).

### 2.2 Definition of SII and diabetes

Diabetes mellitus patients satisfied one or more of the following requirements (21): (1) Fasting blood glucose level of 7.0 mmol/L and above, or an oral glucose tolerance test result greater than or equal to 11.1 mmol/L hours after two hours; (2) Random blood glucose greater than or equal to 11.1 mmol/L; (3) Glycohemoglobin (HbA1c) level greater than or equal to 6.5%; (4) use of insulin or diabetic medicines; and (5) diabetes self-reported as diagnosed by a medical professional.

A routine blood test called a complete blood count is used to identify several illnesses and evaluate a person’s general health. The Beckman Coulter technique is used to calculate a full blood count. SII is computed use platelet× neutrophil/lymphocyte. It was first proposed by Bo Hu et al. in 2014 for assessing the prognosis of hepatocellular carcinoma (8).

### 2.3 Mortality outcomes of the study population

We used specific study identifiers (SEQN) and probability matching to the National Death Index (NDI) as of December 31, 2019, to ascertain the mortality status of our participants. Apart from the records of deaths due to all causes, we additionally collected mortality data, primarily associated with cardiovascular diseases, under the term “Underlying Leading Cause of Death”.

### 2.4 Covariates

Demographic features include things like age, sex (male and female), ethnic (Mexican Americans, other (Hispanics, non-Hispanic Whites, non-Hispanic Blacks and others), educational attainment (Less than high schoo, high school, College or above), and the household poverty-to-income ratio (PIR) (these ratios are classed as follows: ≤1.0%, between >1 and ≤3.0%, and >3.0%). Body mass index (BMI) was divided into four categories: <18.5, 18.5-24.9, 25.0-30, and ≥ 30kg/m2 (22). Risk variables were chosen from the health questionnaire and included history of hypertension(it was defined as follows: 1)a self-reported history of hypertension; 2) the use of anti-hypertensive medication; 3) a mean systolic blood pressure of at least 130 mmHg and/or a mean diastolic blood pressure of at least 80 mmHg), smoking, and alcohol consumption. The participants were classified into three groups based on their current smoking habits: current (those who smoke over 100 cigarettes per day or on occasion), former (those who have smoked over 100 cigarettes but no longer do so), and never (those who have smoked less than 100 cigarettes in their lifetime). Participants were then divided into two categories: heavy drinkers (>2 drinks for males and >1 drink for women per week) and light or non-drinkers (meaning abstaining from alcohol entirely or consuming no more than one drink per week for women and two drinks per week for men) (23). The laboratory variables were blood cell counts, HbA1c, serum creatinine (Scr), low-density lipoprotein cholesterol (LDL), triglycerides (TG), total cholesterol (TC), blood urea nitrogen (BUN) high-density lipoprotein cholesterol (HDL). In conclusion, the NHANES website provides detailed instructions for collecting blood biochemical measurements. To sum up, comprehensive instructions for gathering blood biochemical measures are available on the NHANES website.

### 2.5 Statistical analysis

We employed survey analysis techniques and adhered to NHANES analytical reporting guidelines to guarantee nationally representative estimates (24). The Student t test and the Mann-Whitney U test were used in this study to describe the variations in continuous variables between the two groups. When evaluating group differences in categorical data, the chi-square test was employed.

Using the “maxstat” package (https://CRAN.R-project.org/package=maxstat), the maximum selection rank statistic was utilized to ascertain the optimal SII cut-off point that was most significantly associated with survival outcomes. Participants were then split up into two groups: high and low SII. Survey-weighted Cox regression analysis was used in the study to evaluate the correlation between SII and cardiovascular and all-cause mortality in people with diabetes. Three models were built with potential confounders taken into account. While Model 1 was left unadjusted Model 2 underwent adjustments for gender,age, ethnicity, educational level, and PIR. BMI, drinking and smoking habits, hypertension, TG, TC, HDL, LDL, Scr, and BUN levels were further adjusted for in Model 3.

In diabetic patients, potential nonlinear relationships between SII and cardiovascular and all-cause death were visualized using smooth curve fitting. The log-rank test was utilized to compare the odds of survival, which were calculated using the Kaplan-Meier technique. Furthermore, we investigated the interaction between SII values and mortality by utilizing subgroups based on age, gender, BMI, drinking status, and smoking status to examine the relationship between the two. The data were examined using licensed statistics (http://www.empowerstats.com) and the statistical software R (http://www.R-project.org). P values indicating statistical significance for differences were less than 0.05.

## 3 Results

### 3.1 Baseline characteristics of participants

**Figure 2:**
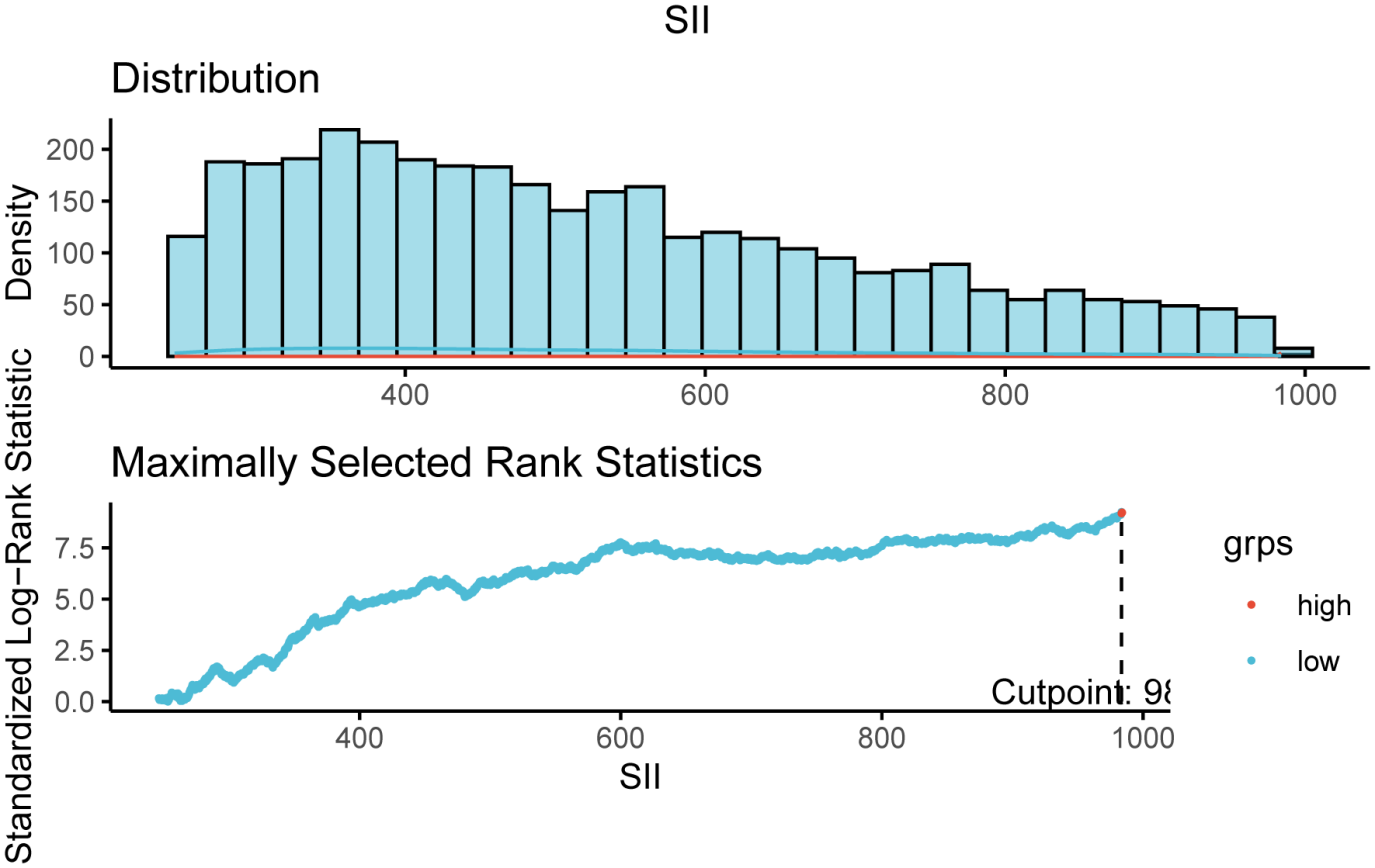
The cutoff point was calculated using the maximally selected rank statistics based on the ‘maxstat’ package.

For this research, a total of 4972 diabetes individuals were included. The maximum selection-based rank statistic (**Figure 2**) was used to determine the ideal SII threshold (983.5714), which divided the participants into two groups: a lower group (SII ≤983.5714, n = 4471) and a higher group (SII >, n = 501). Individuals in the higher SII group were more likely to be white than those in the lower SII group. They also had lower levels of lymphocytes, LDL, TG, TC, HbA1c and higher levels of neutrophils, white blood cells(WBC), Scr, BUN, and BMI. **Table 1** displays further participant characteristics.

**Table 1:** Characteristic of participants.

Variable	Total (n = 4972)	Lower SII (n = 4471)	Higher SII (n = 501)	P value
Age, years	59.03 (58.61,59.45)	58.97(58.53,59.41)	59.58 (58.18,61.01)	0.398
Sex, %				0.001
Female	2396(48.19%)	2120(47.42%)	276(55.09%)	
Male	2576(51.81%)	2351(52.58%)	225(44.91%)	
Race, %				<0.001
Mexican American	917(18.44%)	846(18.92%)	71(14.17%)	
Other Hispanic	507(10.20%)	455(10.18%)	52(10.38%)	
Non-Hispanic White	1862(37.45%)	1603(35.85%)	259(51.70%)	
Non-Hispanic Black	1253(25.20%)	1173(26.24%)	80(15.97%)	
Other Race	433(8.71%)	394(8.81%)	39(7.78%)	
BMI, kg/m2	31.49 (31.29,31.68)	31.45(31.25,31.65)	31.87 (31.12,32.63)	0.206
BMI category, %				<0.001
Lower weight(<18.5)	28 (0.56%)	18 (0.40%)	10 (2.00%)	
Normal (18.5-24.9)	675 (13.58%)	592 (13.24%)	93(18.56%)	
Over weight (25–30)	1458 (29.32%)	1335(29.86%)	123(24.55%)	
Obesity (≥ 30)	2811 (56.54%)	2526(56.50%)	285(56.89%)	
Smoking status, %				0.097
Never	2480(49.88%)	2252(50.37%)	228(45.51%)	
Former	1657(33.33%)	1480(33.10%)	177(35.33%)	
Current	835(16.79%)	739(16.53%)	96(19.16%)	
Drinking status, %				0.694
Never or mild	3946(79.36%)	3545(79.29%)	401(80.04%)	
Heavy	1026(20.64%)	926(20.71%)	100(19.96%)	
Hypertension, %				0.906
Yes	3940(79.24%)	3544(79.27%)	396(79.04%)	
No	1032(20.76%)	927(20.73%)	105(20.96%)	
WBC, × 109/L	7.26(7.20,7.32)	7.08(7.02,7.14)	9.08(8.85,9.30)	<0.001
Neutrophil, × 109/L	4.24 (4.19,4.29)	4.03 (3.99,4.08)	6.63(6.45,6.81)	<0.001
Lymphocyte, × 109/L	2.03 (2.01,2.05)	2.10 (2.08,2.12)	1.48 (1.43,1.53)	<0.001
Platelet, × 109/L	231.85(229.87,233.85)	225.80(223.83,227.79)	293.52(286.16,301.07)	<0.001
Scr, umol/L	82.06 (81.20,82.94)	81.35(80.47,82.24)	88.73(85.25,92.35)	<0.001
BUN, mmol/L	5.16 (5.09,5.22)	5.11 (5.05,5.17)	5.160(5.34,5.88)	<0.001
HbA1c, %	7.05 (7.01,7.09)	7.07 (7.02,7.11)	6.88(6.75,7.02)	0.008
HDL, mmol/L	1.20 (1.19,1.21)	1.20 (1.19,1.21)	1.21(1.18,1.24)	0.617
LDL, mmol/L	2.62 (2.58,2.65)	2.64 (2.60,2.68)	2.46 (2.35,2.57)	0.003
TG, mmol/L	1.50 (1.47,1.54)	1.51(1.48,1.55)	1.44 (1.34,1.54)	0.182
TC, mmol/L	4.73 (4.69,4.76)	4.74 (4.71,4.78)	4.56(4.47,4.65)	<0.001
Education levels, %				0.139
Less than high school	1745(35.10%)	1569(35.09%)	176(35.13%)	
High school	1178(23.69%)	1043(23.33%)	135(26.95%)	
College or above	2049(41.21%)	1859(41.58%)	190(37.92%)	
Family income-poverty ratio, %				0.605
≤ 1.0	1244(25.02%)	1110(24.83%)	134(26.75%)	
1.0–3.0	1458(29.32%)	2038(45.58%)	226(45.11%)	
> 3.0	2811(56.54%)	2252(50.37%)	228(45.51%)	

Continuos variables are presented as the mean and 95% confidence interval, category variables are described as the percentage and 95% confidence interval.

### 3.2 Association of SII with mortality

**Figure 3:**
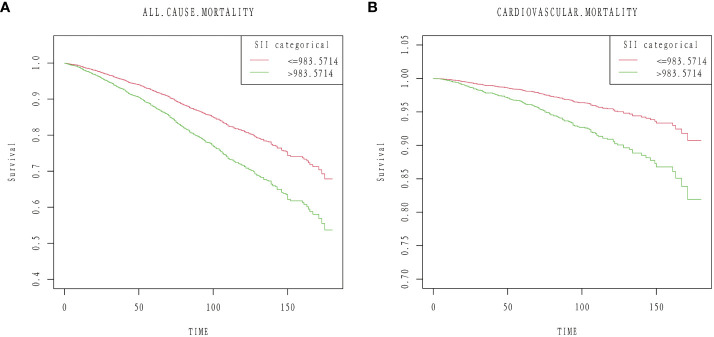
Kaplan–Meier curves of the survival rate: **(A)** All-cause mortality. **(B)** Cardiovascular mortality.

During a median follow-up of 87 months (interquartile range [IQR], 55-124 months), 1,172 (23.57%) of the 4,972 patients with diabetes mellitus passed away. 840 (16.89%) of them were non-cardiovascular fatalities, and 332 (6.68%) were cardiovascular deaths. We discovered that when the SII values increased, there was a substantial increase in the risk of all-cause death in the crude model (HR 2.01; 95% CI:1.73,2.33). After multi-factorial adjustment, there was a corresponding increase in all-cause mortality in patients with diabetes of 81% (model 1, HR 1.81;95% CI:1.56,2.11) and 60% (model 2, HR 1.60; 95% CI:1.29,1.99) for every unit increase in SII value. Furthermore, we discovered that there was a strong correlation between high SII and the risk of cardiovascular death. In the crude model, we discovered that the risk of cardiovascular death rose significantly with rising SII levels (HR 2.41;95%CI:1.85,3.14). Diabetes patients experienced an increase in cardiovascular mortality with each unit increase in SII value of 114% (model 1, HR 2.14;95%CI:1.63,2.80) and 105% (model 2, HR 2.05; 95%CI:1.42,2.97), even after controlling for a number of other variables (**Table 2**). Kaplan-Meier data indicated that the high SII group had a poorer survival rate (**Figure 3**).

**Table 2:** The relationships between SII and mortality in diabetes.

Characteristic	Crude model		Model 1		Model 2	
	HR (95% CI)	P	HR (95% CI)	P	HR (95% CI)	P
All-cause mortality						
SII	1.0005(1.0004,1.0005)	<0.000001	1.0005(1.0004,1.0006)	<0.000001	1.0005(1.0002,1.0006)	0.000045
SII category						
Lower SII(n = 4471)	Ref		Ref		Ref	
Higher SII(n = 501)	2.01(1.73,2.33)	<0.0001	1.81(1.56,2.11)	<0.0001	1.60(1.29.1.99)	<0.0001
Cardiovascular mortality						
SII	1.0006(1.0004,1.0007)	<0.000001	1.0006(1.0004,1.0008)	<0.000001	1.0006(1.0003,1.0009)	0.000064
SII category						
Lower SII(n = 4471)	Ref		Ref		Ref	
Higher SII(n = 501)	2.41(1.85,3.14)	<0.0001	2.14(1.63,2.80)	<0.0001	2.05(1.42,2.97)	<0.0001

Crude Model, unadjusted; Model 1, adjusted for age, sex, race, PIR, educational level; Model 2, adjusted for age, sex, race, BMI, smoking status, drinking status, hypertension, HDL, LDL, TG, TC, education level, BUN and Scr.

### 3.3 Smooth curve fitting

**Figure 4:**
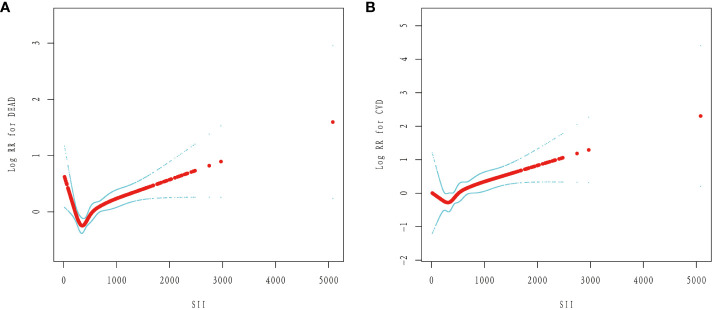
The non-linear relationship between SII and the risk of mortality in diabetes: **(A)** All-cause mortality. **(B)** Cardiovascular mortality.

After fully accounting for covariates, we fitted a smooth curve of SII with cardiovascular and all-cause mortality in diabetics (model 3). In diabetic patients, we discovered a “L” shaped correlation between SII and CVD and all-cause mortality (**Figure 4**).

### 3.4 Subgroup analysis

Additionally, we used subgroup analyses based on gender, age, BMI, drinking, smoking, and hypertension. However, we were unable to determine whether SII and the previously mentioned variables interacted significantly (p interaction > 0.05) (**Table 3**).

**Table 3:** Subgroup analysis of the associations between NLR and mortality among diabetes.

Characteristics	Lower SII	Higher SII	P#	p interaction	Higher SII	P*	p interaction
		HR#(95% CI)			HR*(95% CI)		
Age, y				0.2126			0.0908
< 60	1	1.97(1.13,3.46)	0.0174		4.54(1.62,12.71)	0.0039	
≥ 60	1	1.53(1.22,1.93)	0.0003		1.96(1.32,2.91)	0.0008	
Sex				0.1717			0.1692
Female	1	1.25(0.90,1.74)	0.1824		1.52(0.86,2.69)	0.1473	
Male	1	1.79(1.35,2.39)	<0.0001		2.68(1.65,4.36)	<0.0001	
Smoking status				0.4078			0.6919
Never	1	1.46(1.05,2.03)	0.0257		1.81(1.04,3.16)	0.0374	
Former	1	1.82(1.31,2.52)	0.0003		2.62(1.48,4.63)	0.0009	
Current	1	0.87(0.49,1.54)	0.6365		1.23(0.43,3.54)	0.6995	
Drinking status				0.2688			0.0802
Never/Mild	1	1.52(1.21,1.91)	0.0003		1.78(1.18,2.69)	0.0057	
Heavy	1	1.75(0.91,3.36)	0.0952		3.78(1.52,9.40)	0.0042	
Hypertension				0.9056			0.4576
Yes	1	1.58(1.26,1.98)	<0.0001		2.09(1.42,3.08)	0.00002	
No	1	1.07(0.56,2.02)	0.8460		1.23(0.27,5.51)	0.7872	
BMI, kg/m2				0.4791			0.9629
Lower weight(<18.5)	1	1.07(0.01,2.02)	0.5825		0.01(0.01,5.51)	0.9970	
Normal (18.5-24.9)	1	2.08(1.34,3.24)	0.0012		1.36(0.54,3.40)	0.5113	
Over weight (25–30)	1	1.34(0.86,2.09)	0.2011		2.48(1.25,4.95)	0.0098	
Obesity (≥ 30)	1	1.52(1.13,2.06)	0.0059		2.34(1.42,3.86)	0.0008	

#All-cause mortality; *cardiovascular mortality. HRs were adjusted for adjusted for age, sex, race, BMI, smoking, drinking, hypertension, HDL, LDL, TG, TC, education level, family income-to-poverty ratio, BUN and Scr

## 4 Discussion

The prevalence of diabetes has dramatically expanded in tandem with the expansion of the global economy. A number of chronic comorbidities and problems brought on by diabetes drastically shorten life expectancy (1). Therefore, reducing diabetes complications and improving patient quality of life can be achieved by having a complete grasp of the underlying causes that influence the development of lesions. Previous studies have shown a 4% increase in the likelihood of developing diabetes for each unit increase in SII. However, there are fewer studies evaluating SII scores and the prognosis of diabetic patients, and the aim of this study was to evaluate the relationship between SII scores and mortality in diabetic patients (18).

There are not many studies on SII and diabetes prognosis. In 4,972 diabetic patients from six NHANES cycles (2005-2016), our analysis demonstrated that increased SII was substantially linked with all-cause and cardiovascular mortality. The connection held true even after controlling for covariates. Furthermore, in individuals with diabetes, smooth curve fitting demonstrated a non-linear connection between SII and cardiovascular and all-cause mortality. To sum up, SII is a reliable indicator for estimating the chances of survival for individuals with diabetes. This screening method can be used to rapidly identify people who are at a high risk of dying or suffering from unfavorable health outcomes at a relatively low cost.

Insulin resistance is associated with immune system activation and persistent low-grade inflammation, and it is connected to both obesity and type 2 diabetes mellitus (T2DM) (25). Risk factors for the onset of T2DM and its macrovascular consequences include systemic inflammatory indicators.Numerous prospective and cross-sectional studies have reported elevated levels of cytokines and chemokines, sialic acid, and circulating acute-phase proteins (such as, fibrinogen, haptoglobin, C-reactive protein (CRP), fibrinogen activator inhibitor, and serum amyloid A) in patients with type 2 diabetes. Moreover, increased CRP, interleukin-1β (IL-1β) and IL-6 T2DM-predictive values are found (11, 26–28). Based on the available data, diabetes is thought to be influenced by inflammation. Furthermore, a multitude of studies have demonstrated that persistent inflammation can also give rise to a wide range of vascular and diabetic problems (25). In individuals with type 2 diabetes, greater levels of SII were linked to diabetic kidney disease (DKD), according to a cross-sectional study by Shao L et al (29). SII might be a straightforward and affordable way to identify DKD. Zhang J et al. shown in a prospective cohort analysis a substantial correlation between high SII and elevated cardiovascular and all-cause mortality in the overall populace (17). The finding that a greater SII is substantially linked to an increased risk of cardiovascular diseases (CVDs) is supported by a recent meta-analysis (30). Additionally, it has been noted that elevated SII raises the hazard of all-cause death as well as the subtypes of ischemic and hemorrhagic stroke (31). In conclusion, the above evidence suggests that SII is a valid predictor for assessing the prognosis of diabetic complications and CVDs in the general population. SII levels can be used as an easily detectable biomarker of systemic inflammatory activity. They are based on the counts of three circulating immune cells (i.e., neutrophils, lymphocytes, and platelets) and represent the inflammatory state (16, 29). After adjusting for confounders, our study’s findings indicated that diabetes individuals with higher SII had worse overall survival. The optimally selected rank statistic, an outcome-oriented method that yields the cutoff point most substantially associated with survival outcomes, identified the best threshold (983.5714). Furthermore, smoothed curve fitting revealed a substantial nonlinear connection between SII and diabetes-related survival outcomes.

Our study’s huge sample size and long follow-up period allow us to confidently make conclusions, which is only one of its many benefits. Secondly, in order to examine the potential use of a unique inflammatory index for diabetes patient mortality prediction, we adjusted for a wide range of established risk factors to rule out potential confounders. Third, selection bias was avoided because all participants were taken from the NHANES survey, which uses a sophisticated multistage probability sampling technique. There are a few restrictions to be aware of, though. First off, even though we took a number of possible confounders into account when conducting our studies, it is still possible that other unidentified factors had an impact on SII. Secondly, American patients with diabetes participated in this study. To determine whether the results can be applicable to other populations, further research is necessary. Third, while SII is easily measured in clinical settings, it is frequently lost along with neutrophil, lymphocyte, and platelet counts, which might result in bias in the selection process.

## 5 Conclusions

Overall, we analyzed 4972 diabetic patients over 6 cycles (2005-2016) and concluded that (1) high SII levels were significantly associated with all-cause and cardiovascular mortality in diabetic patients, and (2) there was a nonlinear association between SII and both all-cause and cardiovascular mortality in diabetic patients. Our findings emphasize the significance of using SII as a prognostic indicator for diabetes mellitus in daily clinical practice.

## Data availability statement

The original contributions presented in the study are included in the article/supplementary material. Further inquiries can be directed to the corresponding author.

## Author contributions

CM: Conceptualization, Data curation, Investigation, Methodology, Software, Writing – original draft. KL: Conceptualization, Data curation, Funding acquisition, Methodology, Visualization, Writing – review & editing.
